# Microbial biotechnological approaches: renewable bioprocessing for the future energy systems

**DOI:** 10.1186/s12934-021-01547-w

**Published:** 2021-03-02

**Authors:** Praveen C. Ramamurthy, Simranjeet Singh, Dhriti Kapoor, Parul Parihar, Jastin Samuel, Ram Prasad, Alok Kumar, Joginder Singh

**Affiliations:** 1 Interdisciplinary Centre for Water Research (ICWaR), Indian Institute of Sciences, Bangalore, India; 2 Department of Botany, Lovely Professional University, Phagwara, Punjab India; 3 Department of Botany, Mahatma Gandhi Central University, Motihari, Bihar India; 4 School of Plant Sciences, College of Agriculture and Environmental Sciences, Haramaya University, Box-138, Dire Dawa, Ethiopia; 5 Department of Microbiology, Lovely Professional University, Phagwara, Punjab India; 6 Waste Valorization Research Lab, Lovely Professional University, Phagwara, Punjab India

**Keywords:** Biomass, Enzymes, Fermentation, Metabolic engineering, Biofuel

## Abstract

The accelerating energy demands of the increasing global population and industrialization has become a matter of great concern all over the globe. In the present scenario, the world is witnessing a considerably huge energy crisis owing to the limited availability of conventional energy resources and rapid depletion of non-renewable fossil fuels. Therefore, there is a dire need to explore the alternative renewable fuels that can fulfil the energy requirements of the growing population and overcome the intimidating environmental issues like greenhouse gas emissions, global warming, air pollution etc. The use of microorganisms such as bacteria has captured significant interest in the recent era for the conversion of the chemical energy reserved in organic compounds into electrical energy. The versatility of the microorganisms to generate renewable energy fuels from multifarious biological and biomass substrates can abate these ominous concerns to a great extent. For instance, most of the microorganisms can easily transform the carbohydrates into alcohol. Establishing the microbial fuel technology as an alternative source for the generation of renewable energy sources can be a state of art technology owing to its reliability, high efficiency, cleanliness and production of minimally toxic or inclusively non-toxic byproducts. This review paper aims to highlight the key points and techniques used for the employment of bacteria to generate, biofuels and bioenergy, and their foremost benefits.

## Background

Formation of better-quality byproducts from biomass by use of microbes is considered as a significant asset to diminish synthetic chemical progressions, which are mainly costly, toxic, and non-renewable [1]. The rapid decline in the fossil fuels level and the increasing worldwide requirement of energy has demanded the generation of substitute fuels that can displace the traditional fossil fuels to decrease the elevated accumulation of greenhouse gases in the environment. Hence, in present day time the creation of conservative, efficient and ecologically beneficial renewable energy fuels is the main requirement around the globe that signifies the ability to instantaneously substitute the traditional fuels to decrease the adverse climatic issues [2]. Escalating attention has been concentrated on biomass application as a renewable energy resource due to the reduced levels of conventional fossil fuels [3]. Employing multifaceted microbes to produce renewable energy sources from the biomass and biological residues are of greater importance, therefore, focus for the synthesis of different biofuels via microbes has been gradually escalating in the present-day time [4]. This is mainly due to the metabolic multiplicity of various microbes that allows the generation of biofuels from different moieties [2].

**Fig. 1:**
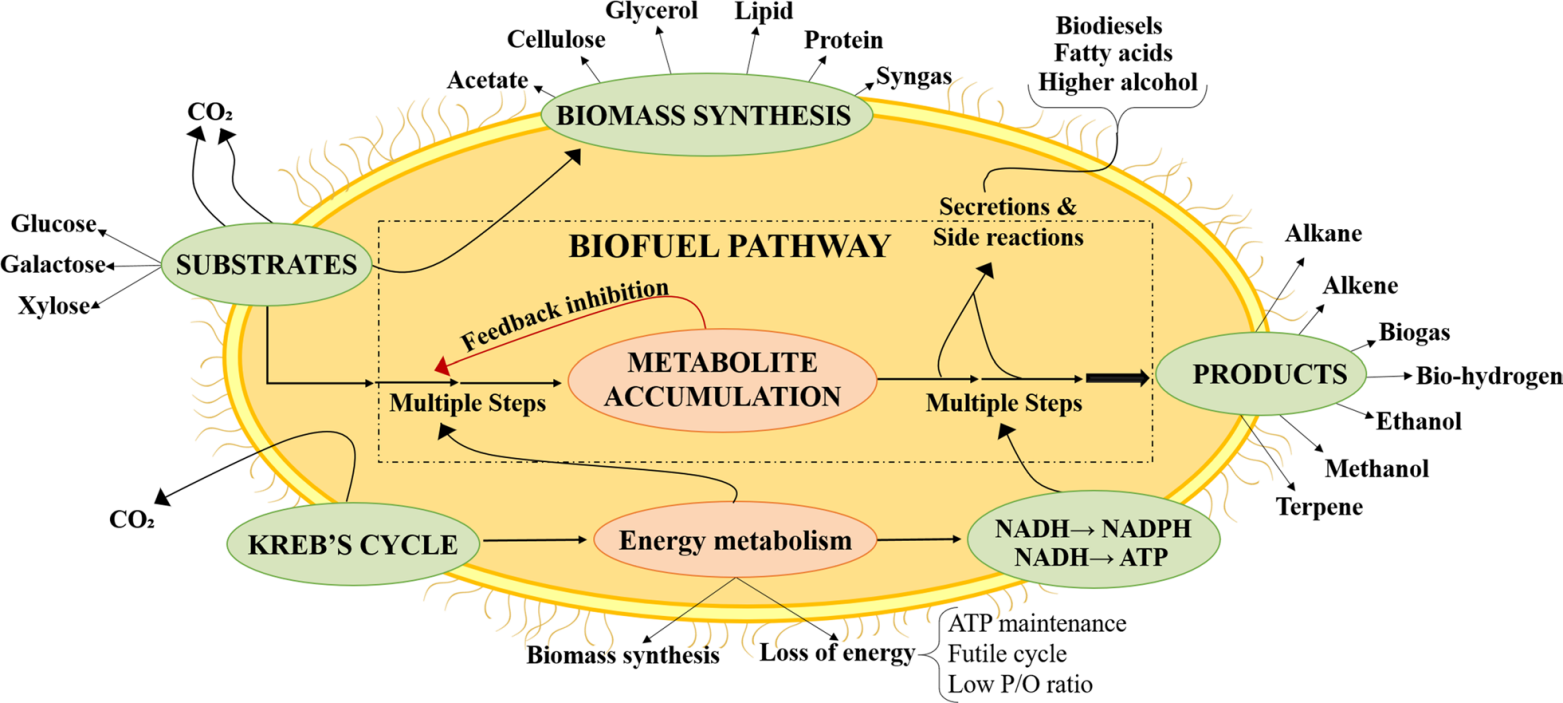
Utilization of renewable feedstocks by microbial cell for the production of diverse bioproducts (Modified from Hollinshead et al. [7])

Microbial biotechnology is an important strategy for sustainable bioprocesses in which microorganisms and their enzymes are used for the conversion of carbohydrates, lignins, glycerols into various renewable resources like bioenergy production (Fig. 1). The buildup of agronomic and industrial wastes in the fields leads to harmful environmental issues. To reduce this problem, microorganisms are of great economic importance in various biotechnological progressions such as in the microorganism fermentative methods. In addition to this, microbes are multifaceted moieties which are helpful in utilization and bioconversion of biomass as they are vital sources of enzymes with biotechnological capability [3]. Microbial glycosylated apparatuses also have significant role to alter the effect of plant contact with harmful pathogens [5] and developing technologies in microbial biotechnology that are beneficial in developing an antimicrobial vaccine and drug discovery in the present-day time [6]. Therefore, this review paper aims to review the various techniques and key points for the generation of bioenergy molecules from microbial functions and their foremost benefits. Moreover, it also focuses on the various metabolic engineering strategies implied to improve the yield of biofuels.

## Microbial biology and energy production

When envisioning the influence of biotechnology in the upcoming time to the energy resources, it is a positive assurance that the generation of biofuels using microorganism may help in providing the energy supply to a greater extent and that the bioprocessing of renewable energy will highly be endorsed by nations where fossil fuel origins are absent [8]. In addition to biodiesel and bioethanol as foremost aspects of biomass energy, H_2_ is also recognized as an efficient and feasible facet for economic and renewable energy resource in the present and future time [9]. In view of energy expenditure prices, biofuel produced from lignocellulosic biomass using *Trichoderma reesei* is an effective and inexpensive method which yields ethanol directly for energy production as it does not need NaClO or acid hydrolysis pretreatment [10, 11]. Microorganisms grouping as consortium instead of using a single micro-organism to elevate the biofuel production may comprise of, (i) *Trichoderma reesei,* for synthesize enzyme to hydrolyze lignocellulosic biomass and *Saccharomyces cerevisiae*, (ii) *Scheffersomyces stipitis*, to exploit hexose and pentose sugars respectively, possibly employed to implement CBP [12]. (iii) Cellulase and xylanase synthesized by *Penicillium echinulatum,* immersed in combined cellulose and sorbitol media, may assist in bioethanol formation from lignocellulosic biomass [13]. (iv) *Anoxybacillus flavithermus* strain TWXYL3 derived xylanase, which is thermostable and alkali stable, can highly contribute to the production of inexpensive, economical and renewable energy [14]. Thermostable enzymes are used as biocatalysts in the biofuel industry; among these, lignocellulose is highly plentiful carbohydrate moiety in the environment, which is ultimately an economical renewable energy asset [15]. (v) *Aspergillus* spp. secretes exo and endo-inulinases enzymes that have been reported to enhance the formation of fructose from inulin and therefore are recognized as promising assets to enhance the biosynthesis of carbohydrates viz., fructose [16].

## Microbial functions in the production of biofuel

Microbial biofuel production is a field where synthetic feedback regulation has the ability for huge influence as biofuels are beneficial alternative energy which may supplement the present-day occurring fuel resources for instance jet fuel, gasoline, or diesel without demanding engine adjustments or extra infrastructure advancement [17]. Inexpensive and efficient strategies are significantly required in the biofuels industry because the major cost is in the manufacture rather than the beginning material phase in this context, consolidated biological processing (CBP) method is considered as the utmost promising method for creating biofuel generation inexpensive in comparison to those biofuels that are presently used commercially [18]. Consolidated biological processing is an effective method which involves one step change of plant materials to biofuels using microbial agents without requiring saccharolytic enzyme supplementation [18]. Nearly 58 bacterial strains, 17 yeasts species and 24 molds have been reported to have the capacity to make bio-ethanol in comparison to other metabolites using several complex metabolic pathways [19]. *Clostridium* species have been engineered to use feedstocks for instance liquefied cornflour [20], glucose [21], glycerol which is then produced during the formation of biodiesel from fats [22] and even syngas which is combined mixture of H_2_ and CO [23] to enhance butanol manufacture. The yeast competence to develop properly on pre-supplemented lignocellulosic biomass could significantly elevate the lipid accretion, which ultimately offers an efficient practice for the manufacture of economically and ecologically sound microbial oil from agronomic residues [24]. Yet till date, * S. cerevisiae* is the most used microorganism for the formation of bio-ethanol because of its greater ethanol productivity, tolerance and competence of fermenting several sugars, in comparison to other microbes [25].

**Fig. 2:**
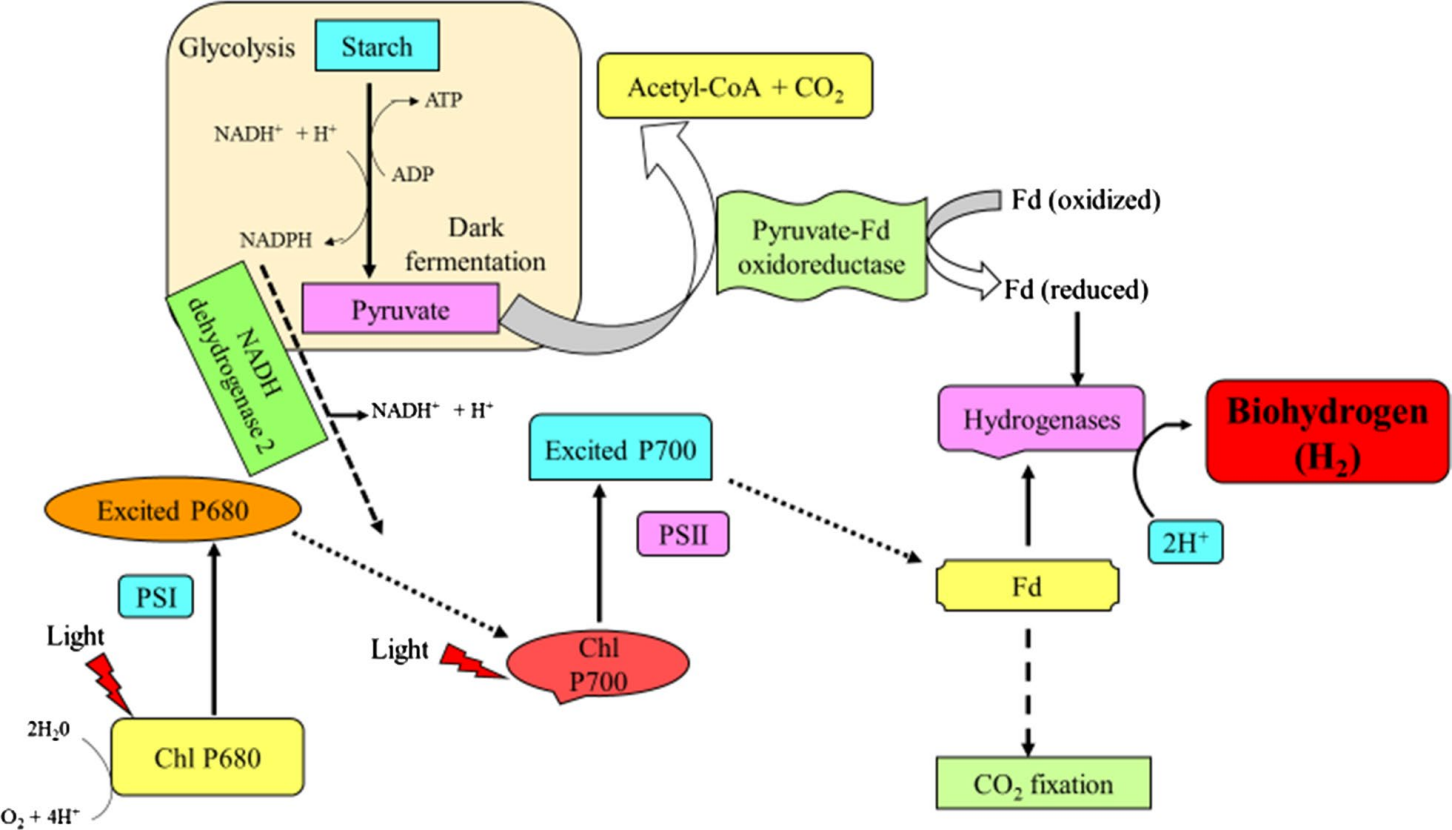
Microbial production of biofuel biohydrogen (modified from Majidian et al. [28]). Chl-chlorophyll; PSI-Photosystem I; PSII-Photosystem II; Fd-ferridoxin; ATP-adenosine triphosphate; ADP-adenosine diphosphate; NADH- reduced form of nicotinamide adenine dinucleotide; NADH^+^- oxidized nicotinamide adenine dinucleotide

Biodiesel production through the use of microbial population such as microalgae, fungi and bacteria are recognized beneficial substitutes for the formation of biodiesel, oil of these oleaginous microorganisms has the potential to be used as the crude moieties for the production of bio-diesel during transesterification [26]. The usage of these quickly developing microorganisms could prove considerably promising as it services a greater form of feedstock such as sugarcane with amazingly bigger product/hectare compared to rape seeds and biological mass, and hence ability to make biodiesel by using a lesser part of arable land [27]. Due to the fast-growing nature, ability to double their biomass within 24 h and extensively rich in oil, microalgae are considered as one of the chief source of biodiesel that has the capacity to partially substitute fossil diesel requirement [28]. *Saccharomyces cerevisiae* CHY1011 improved production of bioethanol via employing alkali-treated *Miscanthus sacchariflorus* as carbon source [29]. Oils can also be produced from rapidly growing microorganisms followed by transesterification via simple chain alcohols, therefore forming an economical superior biodiesel ester that succeeds with current standards [30]. A2A an *E. coli* strain which uses glucose or hemicellulose to form fatty acids can be employed for biodiesel production [31]. Microbes like blue green algae, a few dark fermenting microbes, and purple non-sulfur photosynthetic bacteria have also been employed in the production of biohydrogen [32] (Fig. 2). CH_4_ from a landfill or natural gas wells which have otherwise poor performance can be employed straightforwardly by methanotrophs to make fuels, or can be changed to methanol (CH_3_OH) and ultimately used by methylotroph organisms for fuel generation [4], these microbes oxidize CH_4_ by firstly starting reduction of oxygen molecules to hydrogen peroxide and after that conversion of CH_4_ to CH_3_OH using CH_4_ monooxygenases [33].

## Metabolic engineering for biofuel production

Advancement in the field of the metabolic engineering has accelerated the production of biofuels like fatty acid, alcohols, and gaseous derivatives may have the growing possibility to compete with the fossil fuels being used these days. The following sub-sections, will have discussions on the procedure which is taken into consideration for metabolic engineering. Compilation of the studies showing the metabolic engineering strategies implied to improve the yield of biofuels has been cited in Table 1.

**Table 1:** Compilation of the studies showing the metabolic engineering strategies implied to improve the yield of biofuels

Classes	Biofuel	Microorganisms	Carbon source	Metabolic engineering strategy	Yield	References
Alcohol based products	Ethanol	*Synechocystis* sp. PCC 6803	Glucose	Double homologous recombination technology was used to integrate the alcohol dehydrogenase II (*adh*) and pyruvate decarboxylase (*pdc*) genes from *Zymomonas mobilis* into the *Synechocystis* PCC 6803	5.2 mmol OD_730_ /unit/L/day	[34]
	Ethanol	*Synechococcus* sp. PCC 7942	Glucose	Transformation of *Synechococcus* sp. strain PCC 7942 using the bacterium *Zymomonas mobilis* by cloning the coding sequences of alcohol dehydrogenase II (adh) and pyruvate decarboxylase (pdc) into the shuttle vector pCB4.	6 mmol OD_730_/unit/L/day	[35]
	Ethanol	*Synechocystis* PCC 6803	Glucose	Engineering the pathway involving ribulose-1,5-bisphosphate carboxylase/oxygenase (RuBisCO), fructose-1,6/sedoheptulose-1,7-bisphosphatase (FBP/SBPase), transketolase (TK) and aldolase (FBA)	Enhanced by 43.6% for EtOH-rbcSC, 45.2% for EtOH-70glpX, 38.4% for EtOH-tktA and 47.4% for EtOH-fbaA.	[36]
	Butanol	*Clostridium acetobutylicum*	Glucose	The *pta* and *buk* genes were disrupted that encoded for phosphotransacetylase and butyrate kinase, while the gene *adhE1*^*D485G*^ encoding for aldehyde/alcohol dehydrogenase, was overexpressed	0.71 mol butanol/mol glucose	[37]
	Butanol	*Clostridium tyrobutyricum*	Lactose	*cat1 gene* responsible for production of *butyrate* was replaced by *adhE1* or *adhE2*	26.2 g/L	[38]
	Butanol	*Clostridium acetobutylicum*	Glucose	AAD (aldehyde/alcohol dehydrogenase) variants using substrate specificity feature analysis, random mutagenesis, and structure-based butanol selectivity design were prepared	17.47 and 15.91 g butanol/g ethanol for AAD_F716L_ and AAD_N655H_	[39]
	Propanol	*Escherichia coli*	Glucose	Deletion of rpos gene and overexpression of cimA and ackA gene that encodes for citramalate synthase and acetate kinase A/propionate kinase II, by introducing a modified adhE gene	0.107 g/g and 0.144 g/L/h^−1^	[40]
	Propanol	*Escherichia coli*	Glycerol	Deletion of rpos gene and overexpression of cimA and ackA gene that encodes for citramalate synthase and acetate kinase A/propionate kinase II, by introducing a modified adhE gene	0.259 g/g and 0.083 g/L/h	[40]
Fatty acid based products	Triacylglycerol	*Rhodococcus opacus*	Glucose	Deletion of acyl-coenzyme A (CoA) synthetases and over-expression of three lipases with lipase-specific foldase	82.9 g/L	[41]
	Lipid	*Yarrowia lipolytica*	Glucose, glycerol	Overexpression of 148 putative transcription factor	Increase was 90.9% using glucose while 74% using glycerol	[42]
	Free fatty acid	*Yarrowia lipolytica*	Glucose	Approaches were used to increase the NAD(P)H utilization while removing the efficiency to use formic acid using synthetic biology-based approaches	98.9 g/L	[43]
	Free fatty acid	*Rhodococcus opacus*	Glucose	Deletion of acyl-CoA dehydrogenases and overexpression of lipases, foldase, acyl-CoA synthetase and wax ester synthase.	21.3 g/L	[41]
	Alkane	*Rhodococcus opacus*	Glucose	Deletion of acyl-CoA dehydrogenases and alkane-1 monooxygenase and overexpression of lipases, foldase, acyl-CoA synthetase and heterologous acyl-CoA reductase, acyl-ACP reductase and aldehyde deformylating oxygenase	5.2 g/L	[41]
	Free fatty acid	*Saccharomyces cerevisiae*	Glucose	Designing and alteration in fine-tuned NADPH, subcellular metabolic trafficking, and ATP supply, while declining the carbon flux to biomass. Moreover, replacement of lipogenesis metabolism with fermentation process.	33.4 g/L	[44]
	Alkane	*Cupriavidus necator*	Glucose	Hetreologous ferredoxin (Fd)–Fd reductase was overexpressed	1.48 g/L	[45]
Isoprenoid based product	Isoprene	*Escherichia coli*	Glucose	Redox cofactor balancing	0.665 g/L	[46]
	α-Santalene	*Yarrowia lipolytica*	Glucose	Overexpression of *ERG8*, *ERG10*, *ERG12*, *ERG13*, *ERG19*, *ERG20*, *HMG1*, and *tHMG1*	13.31 mg/L	[47]
	Geraniol	*Escherichia coli*	Glycerol	Alternative pathway i.e. isoprenoid alcohol (IPA) pathway was used that focuses on the synthesis and subsequent IPAs phosphorylation.	0.6 g/L	[48]
Gaseous products	Hydrogen	*Thermoanaerobacterium aotearoense*	Rice straw	Deletion of Fd:NADP^+^ oxidoreductase (nfnAB)	0.381–0.419 g/L	[49]
	Hydrogen	*Escherichia coli*	Xylose and Glucose	Deletion in gene ptsG (phosphotransferase system) for as well as ldhA and frdD	0.284 g/L	[50]
	Hydrogen	*Chlamydomonas reinhardtii*	CO_2_	Repression of the psbA (PSII D1 protein gene)	60% enhancement in hydrogen production	[51]

### Host selection and deflecting carbon sources

The selection of an organism is the most important factor for metabolic engineering. Model organisms have always been the target for engineering, however, utilizing the models for exploiting non-model organism is now possible due to the recently developed biological tools like genetic engineering, hybridization, etc. These technology has helped in identifying the organisms that are naturally the overproducers of the biofuels, and when these organisms are engineered, they tend to give likely more yield and productivity. Like oleaginous organisms that are rich in oily substances when engineered lead to higher production of fatty acid-based products like triacylglycerols (TAGs), fatty acid ethyl esters (FAEEs), fatty acid methyl esters (FAMEs), etc. It was observed when the wild type of *Rhodococcus opacus* was engineered using the synthetic metabolic pathways, the recombinant strains generated higher amount of free fatty acids (FFAs), FAEEs as well as long-chain hydrocarbons in comparison to the wild type that produced the only triacylglycerol from glucose [41]. Similar to this, another study by Qiao et al. (2017) also highlighted that enhancing the NADPH pool lead to higher production of FAMEs [52]. The organisms not only are used to produce the fatty acid-based product but also the alcohol-based products like ethanol, butanol. Using genetic engineering approach, the * S. cerevisiae* enhanced the production of ethanol, butanol [53]. Moreover, it is also reported that recombinant *Clostridium* sp. subjected to hot channel process, enhanced the butanol production [37].

Not only engineering the host, but deflecting carbon source also enhances the production of these biofuels like enriching the medium for *S. cerevisiae* with malonyl-CoA, NADPH, Acetyl-CoA and ATP which enhances the production of ethanol as well as FFAs [44].

### Modulating the supply of reducing power

Another approach for enhancing the production includes the modulation in the supply of reducing power like NADPH, NADH, etc. as biofuel production demands a larger amount of reducing power supply [44]. It was observed that engineering the pentose phosphate pathway enriches the NADPH pool that helps in enhancing the production of biofuels as well [54]. Utilizing the given idea Jaroensuk et al. introduced heterologous formate dehydrogenase in the aldehyde-deformylating oxygenase (ADO)-dependent pathway to enrich the NADPH pool that aided in enhanced production of hydrocarbon [43]. However, the introduction of heterologous formate dehydrogenase will lead to the formation of formic acid as a byproduct. Thus, to minimize the formation of byproduct that has no contribution in further production of biofuel, researchers tried to introduce heterologous formate dehydrogenase that can aid to production of aforementioned byproduct that could generate more and more reducing power. Construction of 2,3-butanediol biosynthesis pathway in *Clostridium acetobutylicum* as NADH-compensating module is also reported and this modulation upgraded the butanol formation [55]. Alternate to this, the interconversion between two reducing power can help in enriching the pool of other reducing power, likewise coexpression of ferredoxin (Fd) and Fd reductase genes and knocking down of *nfnAB* genes that encodes for NADH-dependent Fd: NADP^+^ oxidoreductase that reduces NADP^+^ by NADH and Fd, that enriches the Fd pool for production of H_2_ [45]. Similarly, Wiegand et al. in 2018, engineered the Fd and Fd-NADP^+^-oxidoreductase enzyme so that the transfer of electron declines from Fd and thus securing the pool of Fd for higher production of hydrogen [56].

### Biosynthetic enzyme engineering

Some enzymes are required for enhanced production of biofuels and engineering those enzymes in terms of stability, and catalytic activity helps in achieving the targets of higher titer and productivity. Example for such strategies can be understood by the research of Kudo et al. (2019), wherein they had shown that when non-conserved ADO was engineered conversion efficiency of this enzyme was increased for hydrocarbon production. This strategy can be utilized for the enhanced synthesis of biofuels using type I polyketide synthases that has low solubility and high catalytic functioning, through polyketide biosynthetic pathway [57]. *Clostridium acetobutylicum* was also engineered to increase the butanol-to-ethanol ratio. In this study the adhE1 (aldehyde/alcohol dehydrogenase) gene was engineered in *C. acetobutylicum* as such that instead of utilizing acetyl-CoA during acetone–butanol–ethanol fermentation preference would be given to butyryl-CoA and this strategy enhanced the ratio by 5.8-fold (17.47 g butanol/g ethanol). Such a strategy can be combined with the machine learning tools to expedite the engineering of enzymes for superior activity [39].

### Engineering new pathways

Devising new synthetic metabolism helps to facilitate the production of biofuels like isoprene-based products. Bruder et al. (2019) reported the replacement of fatty acid photo decarboxylase with ADO-dependent pathway for conversion of fatty aldehydes to hydrocarbons is more efficient [58]. Moreover, the production of isoprenoids is done using the microbial fermentation but scientists engineered the MEP (2-C-methyl-D-erythritol 4-phosphate) [47] and MVA (mevalonate) pathways for higher production of isopentenyl pyrophosphate (IPP), and dimethylallyl pyrophosphate (DMAPP) [46] that are precursors of isoprenoids and this engineering helped in enhancing the production of farnesene and isoprene. However, in a recent work, the higher flux of same compounds was done using the exogenous supply of isoprenol [59] or prenol compounds [60] and the reason for the addition of these exogenous compound help in enhancing the production of dimethylallyl pyrophosphate (DMAPP) and isopentenyl pyrophosphate (IPP) [48]. These advancements can also be exploited for higher H_2_ production, the major limitation in H_2_ production is the labile nature of the pathway towards molecular oxygen produced during the process of photosynthesis. However, this limitation can be overcome by indirect photolysis, dark and light fermentation. But, there is need for developing some O_2_-tolerant hydrogenases or cytosolic hydrogenases that have an efficient catalytic activity to accelerate biological production of H_2_ [50].

### Alternative low-value carbon source

The newer researches are trying to utilize carbon sources that are cheaper and non-edible and thus trying to find out newer avenues for producing biofuel. Several researchers have utilized some heterologous machinery to facilitate the use of lignocellulosic derivatives like arabinose, cellulose, lignin [61–64]. To utilize the lignocellulosic derivatives, several engineering in protein folding, modification in post-translational system, systematic optimization of medium, genome-scale modeling have been done [41]. Moreover, improvement in the tolerance of host strains towards the toxic components of lignocellulosic derivatives is also another approach that can be taken into consideration. The shuffling of genomes of such microorganisms that can directly convert lignocellulosic derivatives could also aid in enhanced production of biofuel. Apart from engineering the microorganisms, another approach that can be taken into consideration is separation and conversion of lignin to some aromatic compounds. Not only, lignin but glycerol has also gain attention in recent years because it is produced as a byproduct during the transesterification process used for conversion of lipids to biodiesel [65]. There are several microorganisms like *E. coli*, *Klebsiella* spp., *Citrobacter* spp., *Trichosporon* spp*., Clostridium* spp., etc. that can directly utilize glycerol, while strains that cannot utilize the glycerol, heterologous machinery of the phosphorylative pathway (via glycerol-3-phosphate), glycerol transport system and oxidative pathway (via dihydroxyacetone) have been presented [66]. In addition to isoprenes and glycerol, volatile fatty acids (VFAs) that can be obtained from non-sterile, anaerobic fermentation of organic wastes like sewage sludge, animal manure etc., can also be utilized to produce biofuels. Still, there is a lack of knowledge in this context and remains an area of continuous research.

## Conclusion and future perspectives

At present, the most challenging issue is the sustainable utilization of energy and to preserve the valuable assets we need to explore some newer avenues for the production of energy in terms of biofuels, bio-products, etc. These days we have our technology at its verge to find some alternative system, and in this context, we came across some engineering technologies that have paved new pathways for utilizing diverse microorganisms to enhance the production of biofuels. In this article, the role of microorganisms in biofuel production, the strategies/ideas and the processes used for engineering these microorganisms have been discussed. Advancement in a couple of years in the field of the metabolic engineering has accelerated the production of biofuels like fatty acid, alcohols, gaseous derivatives, that has potential to compete with the fossil fuels being used these days. In this review article, we have discussed the recent challenge of energy demands and the technologies/strategies that are being employed to meet those demands.

Although there is a huge advancement in the strategies, as well as tools to understand the production mechanism for biofuels or other products but the paradigm has shifted towards the cost-effective approach and researchers, are still needed in this direction. Synthesis of biofuels require a huge amount of reducing powers for reduction of carbon residues but still in this process, a large amount of CO_2_ is lost and not utilized in ethanol production. We need to minimize the cost that is spent on maintaining the fermentation process because not all the plant biomass is utilized in biofuel production, some biomass goes in vain. Another thing that should be focussed is by utilizing low-value carbon sources that can lessen the burden on edible carbon sources. Such strategies that are being deployed should be worked upon, and this may expedite the replacement of biofuels with the conventional energy sources.
